# Resveratrol protects mitochondrial quantity by activating SIRT1/PGC‐1α expression during ovarian hypoxia

**DOI:** 10.1002/rmb2.12323

**Published:** 2020-03-16

**Authors:** Akemi Nishigaki, Takeharu Kido, Naoko Kida, Maiko Kakita‐Kobayashi, Hiroaki Tsubokura, Yoji Hisamatsu, Hidetaka Okada

**Affiliations:** ^1^ Department of Obstetrics and Gynecology Kansai Medical University Osaka Japan

**Keywords:** hypoxia‐inducible factor‐1α, mitochondrial DNA copy number, PGC‐1α, resveratrol, sirtuin‐1

## Abstract

**Purpose:**

Resveratrol is a well‐known potent activator of sirtuin‐1 (SIRT1). We investigated the direct effects of hypoxia and resveratrol on SIRT1/ peroxisome proliferator‐activated receptor‐gamma coactivator 1α (PGC‐1α) pathways, vascular endothelial growth factor (VEGF), hypoxia‐inducible factor (HIF)‐1α, and mitochondrial quantity in a steroidogenic human ovarian granulosa‐like tumor cell line (KGN) cells.

**Methods:**

KGN cells were cultured with cobalt chloride (CoCl_2_; a hypoxia‐mimicking agent) and/or resveratrol. The mRNA and protein levels, protein secretion, and intracellular localization were assessed by real‐time PCR, Western blot analysis, ELISA, and immunofluorescence staining, respectively. Mitochondrial quantity was measured based on the mitochondrial DNA (mtDNA) copy number.

**Results:**

CoCl_2_ simultaneously attenuated the levels of SIRT1 and mtDNA expression, and induced the levels of VEGF protein production. In contrast, resveratrol significantly increased the levels of SIRT1 and mtDNA copy number, but reduced VEGF production in normoxia. Resveratrol could recover CoCl_2_‐suppressed SIRT1 and mtDNA expression and antagonize CoCl_2_‐induced VEGF production. CoCl_2_ treatment resulted in a downregulation of PGC‐1α expression, and this effect was recovered by resveratrol. Resveratrol significantly suppressed the production of the CoCl_2_‐induced HIF‐1α and VEGF proteins.

**Conclusion:**

These results suggest that resveratrol improves mitochondrial quantity by activating the SIRT1/PGC‐1α pathway and inhibits VEGF induction through HIF‐1α under hypoxic conditions.

## Graphical abstract

Resveratrol improves mitochondrial quantity by activating the SIRT1/PGC‐1α pathway in hypoxia. PGC‐1α was strong and predominantly localized to the nucleus of the cells treated with resveratrol. CoCl2‐reduced PGC‐1α mRNA and protein expression was recovered by resveratrol.

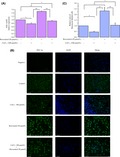

## 1 INTRODUCTION

The oocyte, having no direct vascular supply, is dependent on oxygen diffusion through the surrounding granulosa cells (GCs) and follicular fluid (FF). The development of follicular microvasculature is regulated by angiogenic factors including members of vascular endothelial growth factor (VEGF), epidermal growth factor, and fibroblast growth factor family. These angiogenic factors are produced by GCs and are often secreted or transported into FF.^1^, ^2^, ^3^, ^4^ Friedman et al reported that VEGF was increased in FF of older women and was recognized as a marker of follicular hypoxia.^5^ Hypoxic stress is considered to affect the process of ovarian follicle growth and development. A deficient microvasculature associated with aging around the dominant follicle resulted in hypoxia and a predisposition to increased incidence of the aneuploid oocyte associated with advanced reproductive age.^6^

Cellular hypoxia significantly decreased the expression of mitochondria genes.^7^, ^8^ Mitochondria are multifunctional organelles that are important for energy production, apoptosis, and calcium homeostasis.^9^, ^10^ Mitochondrial number is closely related to oocyte maturation, fertilization, and subsequent development.^11^, ^12^, ^13^, ^14^ The suppression of mitochondrial quantity by aging and hypoxia may be the primary causes of infertility in aged animals.^15^, ^16^

Hypoxia‐inducible factor (HIF)‐1α is stably expressed during hypoxia and is a transcription factor known to play a critical role in the cellular response to hypoxia. HIF‐1α activation in the hypoxic microenvironment contributes to the induction or reduction of the expression of genes involved in different cellular functions, such as angiogenesis, cell survival, oxygen homeostasis, proliferation, glucose metabolism, and apoptosis.^17^, ^18^ For example, HIF‐1 transcriptionally regulates VEGF expression and binds directly to the hypoxia‐response elements in the promoters of the VEGF‐regulated genes.^19^, ^20^

Some flavonoids inhibit the expression level of HIF‐1α protein.^21^, ^22^ The flavonoid resveratrol is a small polyphenol that is found in several plants such as peanuts, berries, and grape skin and thus in red wine.^23^ Resveratrol is a well‐known potent activator of sirtuin‐1 (SIRT1).^24^ SIRT1, a NAD‐dependent protein deacetylase, is controlled by NAD/NADH levels and plays an important role in deacetylation of peroxisome proliferator‐activated receptor‐gamma coactivator 1α (PGC‐1α).^25^, ^26^ PGC‐1α is known as a key transcription coactivator regulating energy metabolism.^27^ Both SIRT1 and PGC‐1α are involved in the mitochondrial biogenesis and inflammatory processes. It has been reported that reduction of SIRT1 activity results in inhibition of PGC‐1α.^28^, ^29^, ^30^ In human GCs, the exact roles of resveratrol in terms of its cytoprotective effects and ability to improve mitochondrial quantity in a hypoxic condition remain unclear.

In the present study, the steroidogenic human ovarian granulosa‐like tumor cell line (KGN) cells were analyzed in vitro, because KGN cells are applicable as a useful model to study steroidogenesis, cell growth, and apoptosis of human granulosa cells. KGN cells are also undifferentiated and maintain the physiological characteristics of ovarian cells, including the expression of functional follicle‐stimulating hormone receptor and the expression of CYP19A1.^31^

In this study, we investigated the direct effects of hypoxia and resveratrol on the SIRT1/PGC‐1α pathways, VEGF, HIF‐1α, and mitochondrial quantity in KGN cells.

## 2 MATERIAL AND METHODS

### 2.1 Cell culture and treatment

The KGN cell line was purchased from the RIKEN Cell Bank of Japan. KGN cells were maintained using Dulbecco's modified Eagle's medium (DMEM)/F‐12 with 10% fetal calf serum (FCS) (HyClone), 100 U/mL penicillin, and 100 μg/mL streptomycin (Invitrogen) in an atmosphere of 5% CO_2_ at 37°C.

KGN cells were seeded into 6‐well plates (1 × 10^6^ cells/well) for real‐time PCR analyses and Western blotting. The cells reached confluence in 2 days and were then used for experiments. KGN cells were cultured in 10% FCS supplemented medium containing various amounts of cobalt chloride (CoCl_2_, a hypoxia‐mimicking agent), resveratrol (Sigma‐Aldrich Corp.), and/or 0.01% DMSO as vehicle control for 6 or 24 hours under 5% CO_2_ in air. The supernatant was collected after stimulation and stored at −80°C until assayed. Each experiment was repeated at least three times with different cell preparations.

### 2.2 Biochemical assay

Concentrations of VEGF in cell culture supernatants were determined with a commercially available enzyme‐linked immunosorbent assay (ELISA) kit (Duoset^®^ ELISA human VEGF, R&D Systems). Intra‐ and inter‐assay coefficients of variation (CVs) in cell culture supernatants were 2.2% and 8.9%, respectively.

### 2.3 RNA extraction and real‐time PCR analysis

Total RNA was isolated from cultured KGN cells using RNeasy Minikit (Qiagen GmbH) according to the manufacturer's instructions. Quatitative real‐time PCR (*q*PCR) was performed using Rotor‐Gene Q HRM (Qiagen) and a quantitative PCR mix kit (THUNDERBIRD SYBR qPCR Mix; TOYOBO), according to the manufacturer's instructions.

*q*PCR was done in a final volume of 20 μL, including 10 μL THUNDERBIRD SYBR qPCR Mix, 4 μL primers (3.75 μmol/L; 2 μL each of both forward and reverse primers), 2 μL cDNA templates, and 4 μL distilled water. Each PCR run was performed as follows: initial denaturation at 95°C for 1 minute, 40 amplification cycles of real‐time fluorescence measurement and denaturation at 94°C for 30 seconds, annealing at 55°C for 30 seconds, and elongation at 72°C for 30 seconds, respectively. Each experiment was performed in duplicate. Elongation factor‐1α (*EF‐1α*) was used as an internal control, as it is a valid reference “housekeeping” gene for transcription profiling, which is also used for real‐time PCR experiments. The primer sets used are described in Table 1.

**Table 1:** Primer sequence used for real‐time PCR and amplicon sizes

Gene	Primer sequence 5′→3′	Product size (bp)
PGC‐1α	Forward: GCT GAC AGA TGG AGA CGT GA	136
	Reverse: TAG CTG AGT GTT GGC TGG TG	
SIRT1	Forward: GCC TCA CAT GCA AGC TCT AGT GAC	97
	Reverse: TTC GAG GAT CTG TGC CAA TCA TAA	
EF	Forward: TCTGGTTGGAATGGTGACAACATGC	329
	Reverse: AGAGCTTCACTCAAAGCTTCATGG	
ND1	Forward: TTC TAA TCG CAA TGG CAT TCC T	109
	Reverse: AAG GGT TGT AGT AGC CCG TAG	
ND5	Forward: TTC ATC CCT GTA GCA TTG TTC G	154
	Reverse: GTT GGA ATA GGT TGT TAG CGG TA	
GAPDH	Forward: CAGAACATCATCCCTGCCTCTAC	251
	Reverse: TTGAAGTCAGAGGAGACCACCTG	

Polymerase chain reaction of all standards and samples was performed using duplicate reactions, after which a melting curve analysis was performed to monitor PCR product purity. To eliminate the possibility of contamination with genomic DNA during extraction of total RNA, a control reaction with each primer pair was performed simultaneously under identical conditions without reverse transcription, and no amplification was detected.

The relative mRNA expression level from real‐time PCR was calculated using the ΔΔthreshold cycle (Ct) method, as described.^32^

### 2.4 Western blot analysis

Cultured cells treated with CoCl_2_ with or without resveratrol were homogenized in lysis buffer containing mammalian protein extraction reagent (Thermo Fisher Scientific Inc) and protease inhibitor cocktail (Calbiochem). The protein concentrations were quantified using Bio‐Rad protein assay reagent (Bio‐Rad Lab.). Equivalent amount of lysate protein (20 μg/lane) were electrophoreses on a 7.5% sodium dodecyl sulfate‐polyacrylamide gel electrophoresis and transferred to Immun‐Blot polyvinylidene diflouride Membrane (Bio‐Rad, Laboratories, Inc). Non–specific‐binding sites were blocked with 10% skim milk powder in Tris‐buffered saline for 1 hour. Blots were then incubated for 1 hour at room temperature (25°C) with rabbit monoclonal SIRT1 antibody (1:400; Santa Cruz Biotechnology, Inc), rabbit monoclonal HIF‐1a antibody (1:1000; Epitomics), or mouse monoclonal β‐actin antibody (1:5000; Sigma‐Aldrich) as the primary antibody, and anti‐rabbit immunoglobulin IgG peroxidase‐labeled secondary antibody (1:10 000; GE Healthcare Life Science) or anti‐mouse IgG peroxidase‐labeled secondary antibody (1:10 000; GE Healthcare Life Science) as the secondary antibody. Immune complexes were visualized using enhanced chemiluminescence plus Western blotting detection reagents (GE Healthcare Life Science). Fold increase was calculated by dividing the relative expression of SIRT1 and HIF‐1α by the relative expression of β‐actin. The protein levels were quantified using Image J.

### 2.5 Measurement of the mitochondrial DNA copy number

Total cellular DNA was isolated from cultured KGN cells using NucleoSpin^®^ Tissue (MACHEREY‐NAGEL GmbH &Co) according to the manufacturer's instructions. Copy number of mitochondrial DNA (mtDNA) was estimated by real‐time PCR analysis using the mitochondrial genes NADH dehydrogenase subunit 1 (*ND1*) and *ND5*. *ND1* and *ND5* levels were normalized to half the level of glyceraldehyde 3‐phosphate dehydrogenase (*GAPDH)* since each cell contains two copies of genomic DNA compared to a single copy of DNA per chromosome. Each sample was run in triplicate, and real‐time PCR analysis was performed as described above. Primer sequences are reported in Table 1.

### 2.6 Immunofluorescence staining

For immunocytochemistry, cells were grown on chamber slides (Thermo Scientific). The medium was removed, and the cells were fixed with 4% paraformaldehyde in phosphate‐buffered saline solution (PBS) for 15 minutes at room temperature. After washing with PBS three times for 5 minutes each, the fixed cells were blocked with 5% normal goat serum and 0.3% Triton X‐100/PBS for 1 hour. Cells were incubated with the primary antibody, PGC‐1α (Abcam: ab54481) diluted in 1% BSA in PBS overnight at 4°C. After washing three times with PBS, cells were incubated for 1.5 hours with Alexa Fluor dye‐coupled anti‐rabbit (Cell Signaling: #4412) secondary antibodies. The unbound secondary antibody was removed with three washes of PBS for 5 minutes each. Next, the samples were counterstained with DAPI (Southern Biotechnology Associates). Samples were visualized on Leica AF7000 fluorescence microscopes. The intensity was quantified using Image J software.

### 2.7 Statistical analysis

Data are expressed as the mean ± standard error of the mean (SEM). Results were analyzed with a statistical software package (StatView II version 4.0; Abacus Concepts). Differences in the measured parameters across the different groups were statistically assessed using analysis of variance (ANOVA) with repeated measurements, followed by Fisher protected least significant difference, multiple range test. A level of *P* < .05 was considered statistically significant.

## 3 RESULTS

### 3.1 Effects of hypoxia on mRNA expression and protein secretion

**Figure 1:**
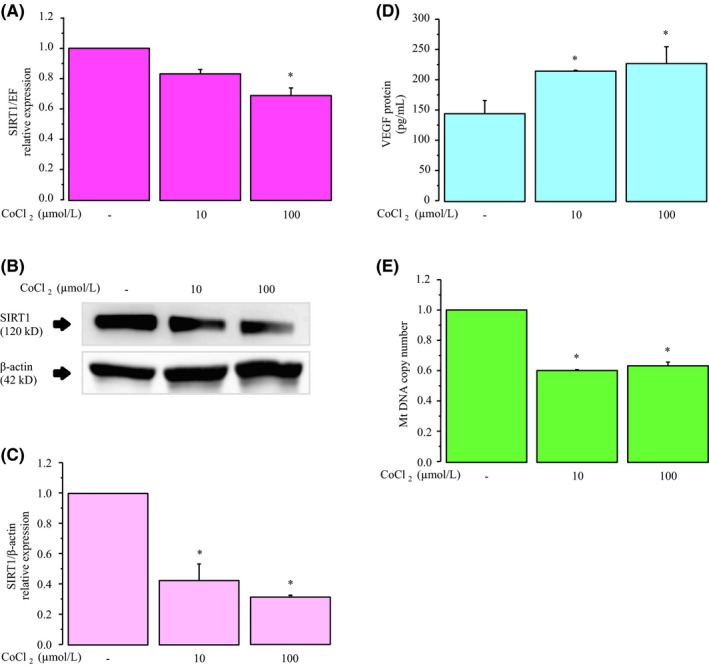
Effects of CoCl_2_‐induced hypoxic stress on mRNA expression and protein secretion. KGN cells were incubated for 24 h in medium containing with 10 μmol/L or 100 μmol/L CoCl_2_. A, *SIRT1* mRNA levels were assessed by real‐time PCR and calculated after normalization to *EF1α* mRNA levels. B, The protein levels of SIRT1 were quantified by Western blotting, and β‐actin was used as the control. C, The protein levels were quantified using ImageJ. D, VEGF protein levels were analyzed by ELISA. E, The mtDNA copy number was determined using real‐time PCR. Fold differences are shown compared with the control, for which the value was defined as 1.0. The data are presented as the mean ± SEM, n = 3. Statistically significant differences are indicated by brackets: *P < .05 versus the control group

We examined the expression of SIRT1 under CoCl_2_‐induced hypoxic stress. As shown in Figure 1A‐C, 100 μmol/L CoCl_2_ significantly attenuated the SIRT1 mRNA and protein expression levels compared with the controls. We next measured VEGF concentration in the culture medium using ELISA. As shown in Figure 1D, the levels of VEGF production were significantly induced by 10 µmol/L and 100 µmol/L CoCl_2_. To confirm the effect of hypoxic stress on mitochondria, mitochondrial quantity was determined by mtDNA copy number. Notably, treatment with 10 µmol/L and 100 µmol/L CoCl_2_ resulted in a downregulation of mtDNA (Figure 1E).

### 3.2 Effect with various concentrations of resveratrol in KGN cells

**Figure 2:**
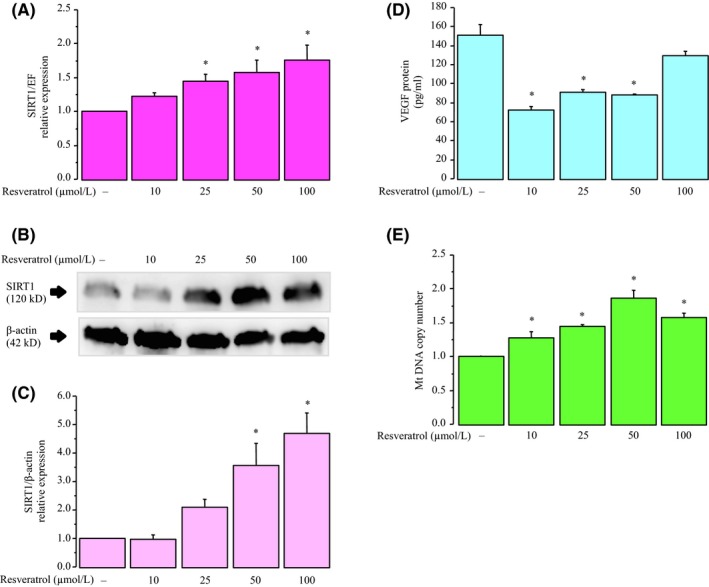
Effects of various concentrations of the resveratrol on KGN cells. Cells were incubated with resveratrol at 0, 10, 25, 50, and 100 μmol/L for 24 h. A, *SIRT1* mRNA levels were assessed by real‐time PCR and calculated after normalization to *EF1α* mRNA levels. B, The protein levels of SIRT1 were quantified by Western blotting, and β‐actin was used as the control. C, The protein levels were quantified using ImageJ. D, VEGF protein levels were analyzed by ELISA. E, The mtDNA copy number was determined using real‐time PCR. Fold differences are shown compared with the control, for which the value was defined as 1.0. The data are presented as the mean ± SEM, n = 3. Statistically significant differences are indicated by brackets: *P < .05 versus the control group

Resveratrol significantly increased SIRT1 mRNA and protein expression in a dose‐dependent manner after 24 hours of stimulation (Figure 2A‐C). In contrast, VEGF protein levels in the culture medium significantly decreased in response to resveratrol at concentrations of 10, 25, and 50 μmol/L (Figure 2D). Resveratrol significantly increased the mtDNA copy number and the highest expression was observed at 50 μmol/L (Figure 2E). Therefore, we chose the 50 μmol/L concentration of resveratrol in all subsequent experiments.

### 3.3 Protective effects of resveratrol against CoCl_2_‐induced hypoxic stress

**Figure 3:**
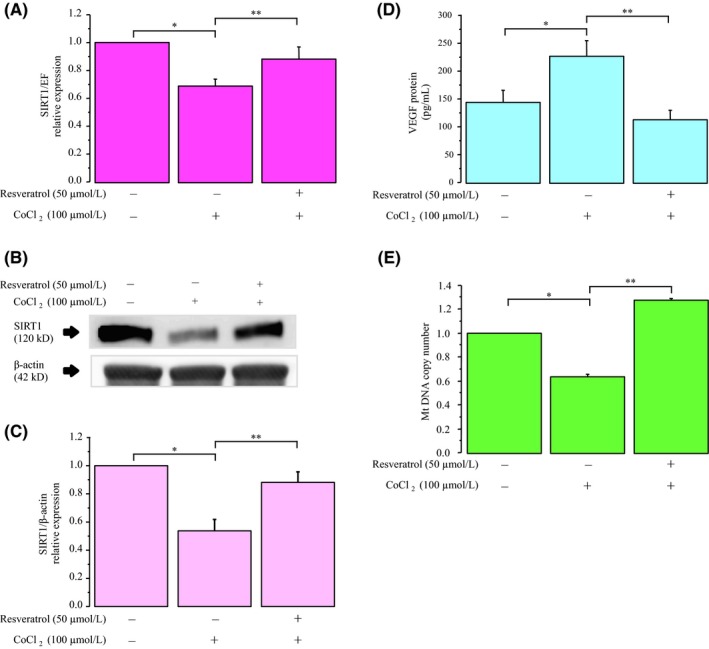
Protective effects of resveratrol against CoCl_2_‐induced hypoxic stress. KGN cells were cultured in medium containing 100 μmol/L CoCl_2_ with or without the 50 μmol/L resveratrol. A, *SIRT1* mRNA levels were assessed by real‐time PCR and calculated after normalization to *EF1α* mRNA levels. B, The protein levels of SIRT1 were quantified by Western blotting, and β‐actin was used as the control protein. C, The protein levels were quantified using ImageJ. D, VEGF protein levels were analyzed by ELISA. E, The mtDNA copy number was determined using real‐time PCR. Fold differences are shown compared with the control, for which the value was defined as 1.0. The data are presented as the mean ± SEM, n = 3. Statistically significant differences are indicated by brackets: *P < .05 versus the control group; **P < .05 versus the 100 µmol/L CoCl_2_ treatment group

To examine the protective effect of resveratrol under CoCl_2_‐induced hypoxic stress, KGN cells were cultured in medium containing 100 μmol/L CoCl_2_ with or without 50 μmol/L resveratrol. As shown in Figure 3A‐C, culture under hypoxic stress resulted in a downregulation of SIRT1 mRNA and protein expression. Resveratrol could reverse the CoCl_2_‐induced inhibitory effect. In contrast, the levels of VEGF protein in the culture medium significantly increased in response to hypoxia, and this effect could be antagonized by treatment with resveratrol (Figure 3D). Additionally, culture under hypoxic stress resulted in a downregulation of mtDNA, and resveratrol was able to recover this decrease (Figure 3E).

### 3.4 Effects of hypoxia and resveratrol on the expression of PGC‐1α mRNA and protein

**Figure 4:**
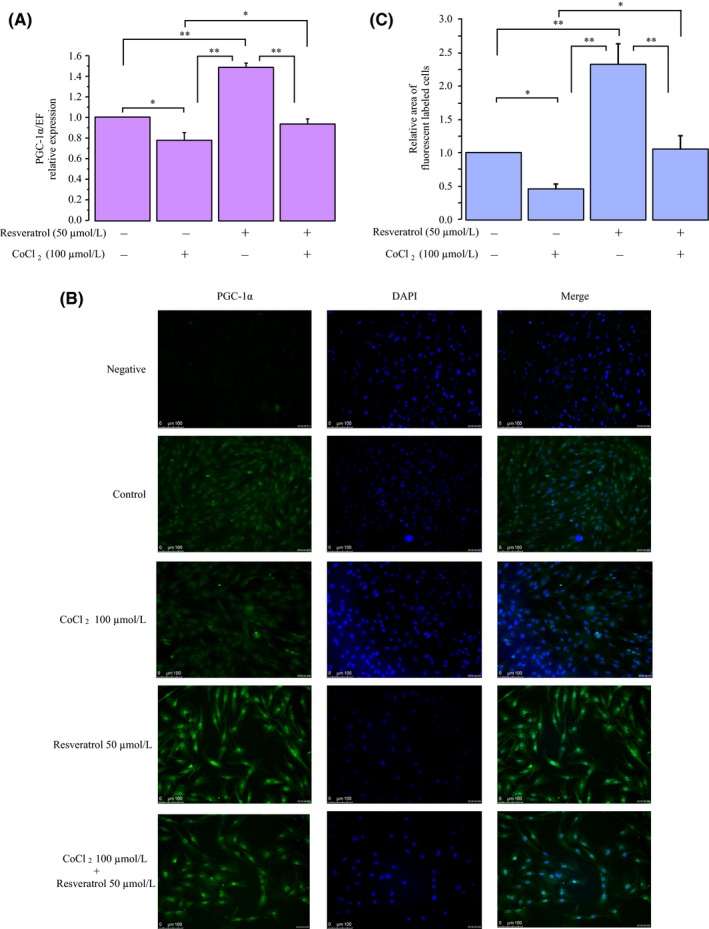
Effects of hypoxia and resveratrol on expression of PGC‐1α mRNA and protein. KGN cells were cultured in medium containing 100 μmol/L CoCl_2_, 50 μmol/L resveratrol, and 100 μmol/L CoCl_2_ plus 50 μmol/L resveratrol for 24 h. A, *PGC‐1α* mRNA levels were assessed by real‐time PCR and calculated after normalization to *EF1α* mRNA levels. B, Immunofluorescence images of PGC‐1α (green) and DAPI (blue) stained cells. C, The protein levels were quantified using ImageJ. Fold differences are shown compared with the control, for which the value was defined 1.0. The data are presented as the mean ± SEM, n = 3. Statistically significant differences are indicated by brackets: ^a^ *P* < .05 versus the control group; ^b^ *P* < .05 versus the 100 μmol/L CoCl_2_ treatment group; and ^c^ *P* < .05 versus the 50 μmol/L resveratrol treatment group

KGN cells were cultured in medium containing 100 μmol/L CoCl_2_, 50 μmol/L resveratrol, and 100 μmol/L CoCl_2_ plus 50 μmol/L resveratrol. As shown in Figure 4A,C, culture under hypoxic stress resulted in a downregulation of PGC‐1α mRNA and protein expression. Resveratrol significantly increased the levels of PGC‐1α mRNA and protein expression similar to SIRT1. Immunofluorescence staining for PGC‐1α was strong and predominantly localized to the nucleus of the cells treated with resveratrol (Figure 4B). Moreover, CoCl_2_‐reduced PGC‐1α mRNA and protein expression was recovered by the 50 μmol/L concentration of resveratrol.

### 3.5 Effect of resveratrol on CoCl_2_‐induced HIF‐1α protein

**Figure 5:**
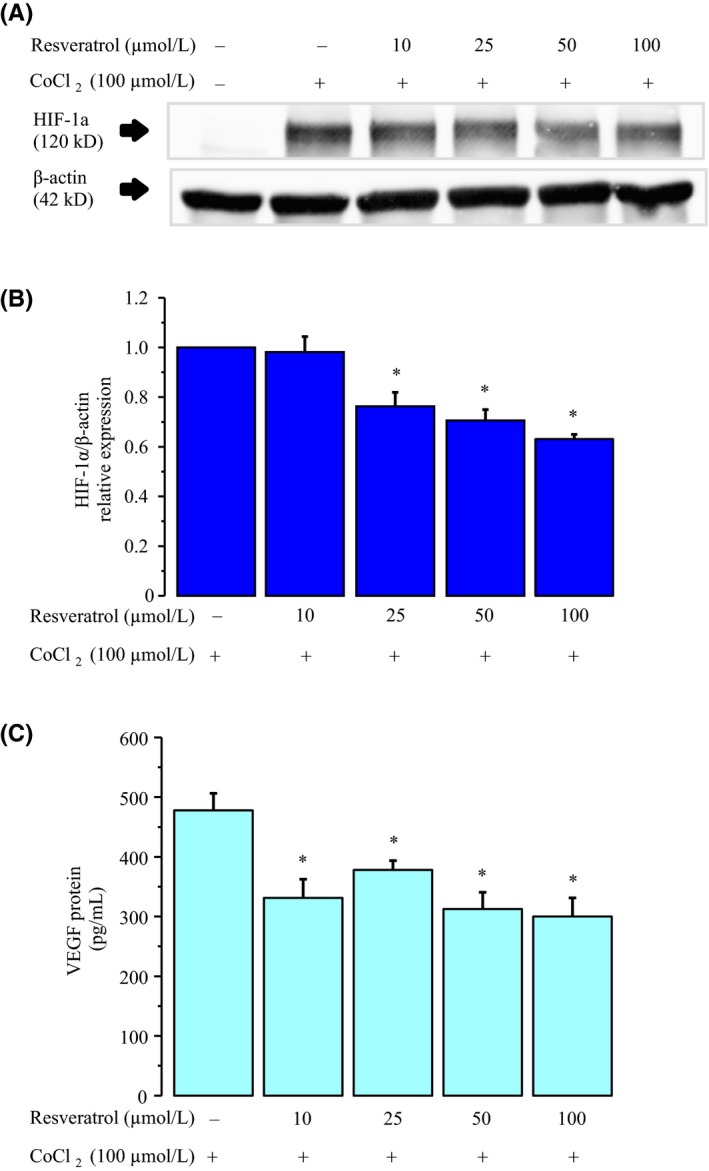
Effect of resveratrol on HIF‐1α. KGN cells were incubated for 24 h in medium containing 100 μmol/L CoCl_2_ with or without 50 μmol/L resveratrol (n = 3). A, The expression of HIF‐1α was quantified by Western blotting. The levels of HIF‐1α were normalized to levels of β‐actin (n = 3). B, The protein levels were quantified using ImageJ. C, VEGF protein levels were analyzed by ELISA. Fold differences are shown compared with the control, for which the value was defined 1.0. The data are presented as the mean ± SEM, n = 3. Statistically significant differences are indicated by brackets: *P < .05 versus the 100 µmol/L CoCl_2_ treatment group

CoCl_2_ can mimic HIF‐1 activation through inhibition of HIF‐1α degradation and the highest expression of HIF‐1α protein was observed at 6 hours, as described previously.^33^ Using Western blot analysis, we determined whether the addition of CoCl_2_ led to an increase in the levels of HIF‐1α proteins at 6 hours in KGN cells. As shown in Figure 5A,B, HIF‐1α protein significantly increased in response to hypoxia. This induction was significantly suppressed by treatment with resveratrol in a dose‐dependent manner. In addition, resveratrol attenuated CoCl_2_‐induced VEGF production in a dose‐dependent manner (Figure 5C).

## 4 DISCUSSION

**Figure 6:**
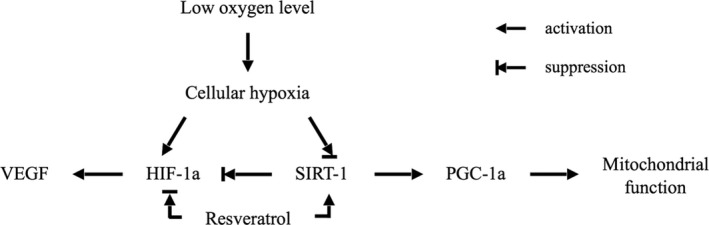
Schematic illustration of the possible mechanism underlying hypoxic stress in KGN cells. Cellular hypoxia inhibits SIRT1 protein and induces HIF‐1α stabilization by inhibition of SIRT1. Moreover, hypoxic stress resulted in a downregulation in PGC1 and mtDNA copy number. In this pathway, resveratrol mediates SIRT1 and mtDNA quantity

In this study, we demonstrated that cellular hypoxia inhibits SIRT1 protein and induces HIF‐1α stabilization in KGN cells. In addition, hypoxic stress resulted in a downregulation of PGC1 and mtDNA copy number, which are likely regulated by SIRT1. Our results suggest that resveratrol improves mitochondrial quantity by activating the SIRT1/PGC‐1α pathway and inhibits VEGF induction through HIF‐1α under hypoxic conditions (Figure 6).

Resveratrol is a well‐known potent activator of SIRT and activates its downstream molecules.^24^, ^34^, ^35^ There have been many studies using several cancer cell lines and animal models that focus on the pleiotropic biological activities of SIRT, including its antioxidant stress, anti‐inflammatory, and anti‐tumor activities.^36^, ^37^, ^38^ SIRT1 expression was observed in the nuclei of GCs at various stages of follicular development.^39^ In the ovary, resveratrol‐induced SIRT1 could enhance progesterone secretion and luteinization‐related genes.^39^ However, the role of the resveratrol to hypoxic stress in the ovary remains poorly understood.

In the present study, we demonstrated that resveratrol treatment markedly upregulated SIRT1 mRNA and protein in KGN cells. In addition, culture under hypoxic stress resulted in a downregulation of SIRT1 mRNA and protein expression, and this effect could be effectively reversed by treatment with resveratrol. Recent studies have reported that SIRT1 expression was correlated with mitochondrial function, energy metabolism, the process of autophagy, apoptosis, and oxidant stress.^27^, ^40^, ^41^, ^42^

SIRT1 and PGC‐1α are well‐known transcription factors, which play pivotal roles in intercellular energy metabolism and gene regulation signal.^29^ Our study demonstrated that the levels of PGC‐1α mRNA and protein expression were increased with resveratrol treatment and culture under hypoxic stress resulted in a downregulation in the expression. The suppression of PGC‐1α expression by hypoxic stress reversed in KGN cells cotreated with resveratrol. The changes in PGC‐1α expression observed in KGN cells coincided with the alternations in SIRT1. These results suggested that SIRT1 influences the expression of its target PGC‐1α. Indeed, several previous studies have demonstrated that SIRT1 can enhance PGC‐1α activation and make it act as a substrate of deacetylation.^25^, ^26^

In normoxia, resveratrol significantly reduced VEGF production from KGN cells. Furthermore, the levels of VEGF protein significantly increased in response to hypoxia and this effect could be antagonized by treatment with resveratrol. Our results are consistent with earlier studies, which state that resveratrol attenuates VEGF expression in several human cancer cell lines.^38^, ^43^ Cao et al showed that resveratrol inhibited hypoxia‐induced VEGF mRNA expression and its protein levels in human ovarian cancer cells in a dose‐dependent manner.^38^ Hypoxia is involved in the regulation the expression of angiogenesis genes such as VEGF. Our findings provide the evidence supporting the anti‐angiogenic effects of resveratrol in hypoxia.

HIF‐1α has been shown to directly bind to hypoxia‐responsive elements in the promoters of the genes encoding VEGF.^19^ Therefore, we presume that HIF‐1α could be a target molecule of VEGF expression. Our study demonstrated that CoCl_2_ significantly induced the expression of HIF‐1α protein in KGN cells. Additionally, resveratrol treatment significantly reduced its HIF‐1α protein levels. Our results are in agreement with recent studies describing that some flavonoids and resveratrol directly inhibit the expression of HIF‐1α protein in hypoxic cancer cells.^38^, ^43^ Mitani et al reported that resveratrol significantly reduced the HIF‐1α protein and VEGF mRNA in hypoxic prostate cancer cells.^37^

HIF‐1α was increased by SIRT1 knockdown and decreased by SIRT1 overexpression. In hypoxia, SIRT1 was suppressed by decrease of oxidized nicotinamide adenine dinucleotide levels, which allowed the activation of HIF‐1α.^44^ These results indicate that resveratrol inhibits VEGF expression by decreasing the expression of HIF‐1α protein through its downstream target SIRT1 in hypoxia. The 10 μmol/L dose of resveratrol reduced VEGF expression, but did not suppress HIF‐1α protein expression. Previous studies have shown that hypoxia stimulated the activation of several signaling pathways.^45^, ^46^ These results suggested that resveratrol is involved in a different signaling pathway from HIF‐1α. In addition, increased VEGF production has been observed in ESCs after stimulation with CoCl_2_, a chemical that induces a hypoxia‐like condition by preventing proteasomal degradation of HIF‐1α proteins.^33^

The present study revealed that resveratrol significantly increased mtDNA copy number. Culture under hypoxic stress resulted in a downregulation of mtDNA, and resveratrol was able to recover this decrease. Interestingly, the change in mtDNA copy number after treatment with resveratrol and CoCl_2_ corresponds with that of *SIRT1* and *PGC‐1α* mRNA expression. PGC‐1α is downstream of resveratrol‐induced SIRT1 and is a central inducer of mitochondrial biogenesis. PGC‐1α can regulate key mitochondrial genes that contribute to play a remarkable role in resistance to oxidative stress.^29^, ^47^, ^48^ These findings support our results that the regulation of SIRT1 and PGC‐1α is closely related to mitochondrial number.

The present study demonstrates that resveratrol enhances SIRT1 expression and mitochondrial function under hypoxic conditions. This finding indicates that resveratrol exerts protective effects against hypoxic stress in KGN cells and acts through the SIRT1/PGC‐1α signaling pathway. It has been reported that decreases in mitochondrial quantity with age may be the primary cause of infertility. Our results suggested that resveratrol may prevent mitochondrial dysfunction due to hypoxia or aging and that resveratrol treatment may be a potential therapy for treating infertility.

## CONFLICT OF INTEREST

The authors declare no conflict of interest.

## HUMAN AND ANIMAL RIGHTS

All the procedures were followed in accordance with the ethical standards of the institutional ethical committee on human experimentation (institutional and national) and with the Helsinki Declaration of 1964 and its later amendments. Informed consent was obtained from all the patients who underwent IVF treatment in the study. This study was approved by the Institutional Review Board at Kansai Medical University. This article does not contain any study that was performed by any of the authors that included animal participants.
